# Validation of the Chinese version of the diabetes health profile to predict the impact of mobile health education on quality of life in type 2 diabetes patients

**DOI:** 10.3389/fpubh.2024.1330154

**Published:** 2024-02-21

**Authors:** Xiaokang Lyu, Jinmei Zeng, Jingna Lin, Yixuan Song, Tingting Yang, Wenjing Hou

**Affiliations:** ^1^Department of Social Psychology, Nankai University, Tianjin, China; ^2^Department of Endocrinology, Health Management Center, Tianjin Union Medical Center, Nankai University Affiliated Hospital, Tianjin, China

**Keywords:** type 2 diabetes, diabetes health profile-18, reliability, validity, quality of life, mobile health

## Abstract

**Purpose:**

The Diabetes Health Profile (DHP18), initially created in the United Kingdom, currently lacks a Chinese version. This study endeavors to authenticate the Chinese adaptation of the DHP18 and assess the influence of mobile health (mHealth) education intervention on the quality of life of individuals living with diabetes.

**Patients and methods:**

The study included 470 Type 2 diabetes Mellitus (T2DM) patients (204 men, 266 women), spanning an age range of 19–79 years, with an average age of 54 ± 12.40 years. Data analysis employed Jamovie and Mplus software. Moreover, test–retest reliability was evaluated in 52 hospitalized T2DM patients through two repeated measurements taken 4 weeks apart.

**Results:**

The Chinese version DHP18 scale exhibited high reliability, evidenced by a Cronbach’s alpha of 0.88, and coefficient of test–retest reliability of 0.84. Individual subscales also demonstrated strong reliability, ranging from 0.76 to 0.84, with test–retest reliability spanning from 0.71 to 0.74. Confirmatory Factor Analysis (CFA) employing a three-factor structure (χ^2^ = 294.69, GFI = 0.92, TLI = 0.91, RMSEA = 0.05, SRMR = 0.06) validated the scale’s construct validity. Notably, there was a statistically significant difference (*p* < 0.05) in the quality of life between Type 2 diabetes patients using mHealth education intervention and those without mHealth education intervention. Mediation analysis revealed that Appraisal of Diabetes (ADS) and Self-Management Efficacy (SED) mediated the effects of Psychological Distress (PD) and Behavior Adherence (BA) on quality of life, both significant direct and indirect effects (*p* < 0.001). In addition, Dietary Abstinence (DE) displayed significant overall impact (*β* = −0.13, *p* < 0.001) and indirect influence (*β* = −0.10, *p* < 0.01) on diabetic patients’ quality of life, though lacking a significant direct effect (*β* = −0.03, *p* = 0.38).

**Conclusion:**

The Chinese version of the Diabetes Health Profile Scale meets stringent psychometric standards and stands as an appropriate measurement tool for Chinese T2DM patients, maintaining comparable results to the original scale’s structure. The mHealth education intervention yielded a notably positive impact on the quality of life among T2DM patients. Mediation analysis revealed that the three dimensions of the DHP were mediated by Appraisal of Diabetes and Diabetes Self-Management Efficacy, partially mediated by Psychological Distress and Behavior Adherence, and fully mediated by Dietary Abstinence, providing insight into the positive effects of the mHealth model on the quality of life of diabetic patients.

## 1 Introduction

Type 2 Diabetes Mellitus (T2DM) is a chronic metabolic disorder characterized by insulin resistance, relative insulin deficiency, and hyperglycemia (1). In China, the prevalence of diabetes among adults over 18-years-old has surged to 11.20% (2), triggering severe complications such as heart disease (3–5), stroke (4, 6, 7), kidney disease (3, 8), vision impairment (9, 10) and neuropathy (11). Additionally, it heightens the risk of depression (7), anxiety (12) and other mental health challenges (13). Managing diabetes effectively (14) involves routine blood sugar monitoring (15), adopting a balanced diet (16, 17) and maintaining consistent physical activity (16, 18, 19). These factors profoundly impact patients’ overall quality of life (20, 21), encompassing both their physical and emotional well-being.

### 1.1 Quality of life in diabetes mellitus

Quality of life (QoL) is a critical health goal that all health interventions strive toward (21). Patients’ perception of their diabetes self-management profoundly influences their QoL, which was often assessed through Patient Self-Reported Outcomes (PROMs) (22, 23). Within PROMs, the evaluating health-related quality of life (HRQOL) holds paramount importance, and various HRQoL questionnaires are utilized in the realm of T2DM (24). HRQOL encompasses a multifaceted construct, encapsulating an individual’s physical, mental and social well-being (25). Unfortunately, China is still in the exploratory phase in this field (26). it is vital to incorporate international diabetes-related scales into the clinical treatment of diabetes in China and adapt them to the local context.

### 1.2 Diabetes self-care education and assistance

Diabetes Self-management Education and Support (DSMES) play a pivotal role in delivering comprehensive diabetes care (27), aiding patients in understanding and managing their condition, consequently improving health outcomes (28). The Standards for DSMES, which were jointly developed by the American Association of Diabetes Educators and the American Diabetes Association and are updated every 5 years, have proven to enhance the lives of individuals with diabetes through diverse health education initiatives (29). In China, although various diabetes awareness projects exist, yet they often lack coordination and are executed on a limited scale by healthcare professionals (30).

The rapid development of internet and online medical services in the past two decades has fundamentally altered DSMES (31–36). The mobile health (mHealth) model, utilizing mobile and wireless technology encompassing medical and public health services accessible via cellular phones (32), patient monitoring devices (34), personal digital assistants (37), and other wireless devices, aims to optimize health system performance and outcomes through extensive use of Information and Communication Technologies (ICTs). Research has explored the potential of using mobile-based technology to assist individuals with T2DM in maintaining an active lifestyle, enhancing diabetes management, fostering patient-provider communication and offering educational opportunities (27). Implementation of these technologies could empower healthcare providers to tailor patient education, addressing head-on the gaps in the current health education systems.

Notably, a research study conducted in China by Dr. Li Jing and his colleagues underscored the positive physiological and biochemical impacts of the mHealth diabetes management model on individuals with diabetes (34). Based on real-world population data from a clinical electronic health database, they assessed the effectiveness of a mobile-based intervention for glycemic control in patients with T2DM. The research study successfully demonstrated the positive impact of mHealth on blood glucose management. However, its effects on the quality of life among people with diabetes remain uncertain. Further research is essential to confirm whether the mHealth interventions can effectively influence the patient-reported psychosocial outcomes, such as behavioral and activity problems, diabetic misery, dietary inhibition and to comprehend the underlying mechanisms driving these effects.

### 1.3 The present study

The Diabetes Health Profile 18 (DHP18), which was originally developed for the United Kingdom (36), has been translated into over 30 languages and is now a widely used scale for diabetes-related issues (20, 38–40). In western societies, the DHP18 has been carefully tested and is used in multi-national clinical investigations, HRQOL research, general surveys, and medical practice (41). Although the DHP18 was selected as the diabetes-specific outcome measure for the UK’s PROMs pilot program, there was no modified Chinese version that has been previously developed (42).

Designed for the use in T2DM patients, the DHP18 has demonstrated adequate internal reliability, validity, and measurement equivalence across various language groups (43–45). Its popularity is attributed to several key aspects. Firstly, its brevity with only 18 questions lessens respondent burden, fostering greater patient engagement, particularly suitable for busy Chinese hospital settings. Secondly, the DHP18 delves into multiple mental health domains, encompassing Psychological Distress (PD), Barriers To Activity (BTA), and Disinhibited Eating (DE). Thirdly, none of the existing Chinese instruments cover the DE subscale, despite evidence indicating that DE is a common issue for many people with T2DM (46).

Given all these factors, this study aims to validate a Chinese version of this multidimensional and user-friendly diabetes-related scale and predicts the predicting its impact on Quality of Life (QoL) and the mechanisms of action within Dr. Jing Li’s mHealth model (34).

## 2 Materials and methods

### 2.1 Patients

The inclusion criteria for the patients in this study were (i) diagnosed with T2DM, (ii) over 18 years of age and (iii) able to take care of themselves. The data was deleted on account of the following three conditions: short response time (less than 3 min), incomplete questionnaire data and regularity of answers. The study yielded 470 valid responses, with the questionnaire’s validity rate being that of 93.25%. Moreover, 52 T2DM patients who were hospitalized in endocrine wards were selected for retest reliability assessment. This same group of patients underwent repeated measurements at one-month intervals during their hospitalization, serving as the baseline reliability test. All patients were aged between 19 and 79 years, with a mean age of 54 ± 12.40 years.

### 2.2 Procedure

At first, we developed a paper questionnaire containing all translated DHP18 items, which were pretested to make sure that there were no difficulties in understanding the program. Other relevant questionnaires were also used in this study and included basic information about diabetes (see Table 1 for detailed demographic information). Verbal Informed consent was obtained in three locations: endocrinology wards of the Tianjin People’s Hospital, the Diabetes Identification Center and the outpatient clinics. For ease of sampling, the questionnaires were administered by trained staff to guide patients when they had questions. The Department of Psychology, Nankai University Ethical Review Committee approved the survey.

**Table 1:** Patient demographic variables (*N* = 470).

Variable	Classifications	Female	Male	*N* (%)	$\chi^{2}$	*p*
Genders	Female	–	–	266 (43.40)		
	Male	–	–	204 (56.60)	8.18	<0.01
Age (years)	≤45	99	43	142 (30.21)		
	>45	165	163	328 (69.79)	75.20	<0.001
Marriage	Single	13	5	18 (3.83)		
	Married	236	175	411 (87.45)		
	Divorced/widowed	15	21	36 (7.66)	635.00	<0.001
BMI (kg/m^2^)	<18.5	4	3	7 (1.49)		
	18.5 ~ 24	65	70	135 (28.72)		
	24 ~ 28	105	73	178 (37.87)		
	≥28	90	57	147 (31.28)	146.00	<0.001
Capacity to pay for health services	No trouble	176	122	298 (63.40)		
	Troubled	88	79	167 (35.53)	36.90	<0.001
Educational level	Junior and lower	59	69	128 (27.23)		
	Senior	86	82	168 (35.74)		
	Three-year college	53	26	79 (16.80)		
	Bachelors and above	63	22	85 (18.09)	45	<0.001
Household	Live alone	25	21	46 (9.79)		
	Not living alone	238	179	417 (88.72)	297	<0.001
Types of labor	Resting	19	30	49 (10.43)		
	Light labor	194	158	352 (74.90)		
	Medium to heavy labor	53	14	67 (14.32)	370	<0.001
Duration of diabetes (years)	<1	46	49	95 (20.21)		
	1 ~ 5	131	83	214 (45.53)		
	≥5	89	72	161 (34.25)	45.40	<0.001
HbA1C (%)	≤7	74	50	124 (26.38)		
	>7	185	159	344 (73.19)	103.00	<0.001
Regular medical visits	Yes	60	61	121 (25.74)		
	No	204	143	347 (73.83)	109.00	<0.001
Diabetes related therapy	Oral medication	174	126	294 (62.55)		
	Insulin	17	9	24 (5.11)		
	Oral medication and insulin	73	66	137 (29.15)	256.00	<0.001
Diabetes education	Not educated	131	101	232 (49.36)		
	mHealth education	59	36	95 (20.21)		
	Other education	74	67	141 (30.00)	62.30	<0.001

Light labor refers to the physical intensity of types of work such as office workers, teachers, etc. Medium to heavy labor refers to the physical intensity of such types of work as drivers, waiters, construction workers, etc. Other health education includes all forms of education that did not use the remote tracking of the system in this study, e.g., nurse presentations in outpatient clinics or wards, small group teaching, look-and-talk, etc.

### 2.3 Instruments

#### 2.3.1 Diabetes health profile

The DHP18, initially developed by Meadows et al. (43), comprises of 18 items in three categories: 6 items for PD, 7 items for BTA, and 5 items for DE. Each entry within these categories is evaluated on a Likert 4-point scale, with scores ranging from 0 to 3 for each item. To calculate the total score for each category, the raw scores were added together and divided by the sum of the highest theoretical scores for that category (18, 21, and 15, respectively), then multiplied by 100 to yield a score ranging from 0 to 100. The instrument was translated from English to Chinese by a physician from the Department of Endocrinology at Tianjin People’s Hospital, and then was back-translated from Chinese to English by a professor in the Department of Social Psychology of Tianjin Nankai University. The translated Chinese version was pre-tested to ensure the quality of the scale translation.

#### 2.3.2 EuroQol five-dimensional questionnaire

The EQ-5D-3L, an HRQoL assessment tool developed by Brooks (47), has been translated into a Chinese version and is widely adopted in China (48). It comprises a descriptive system encompassing five health dimensions: mobility, self-care, usual activities, pain or discomfort, and anxiety or depression. Within each dimension, there are three levels denoting the degree of problems: no problems (score 1), some problems (score 2) and extreme problems (score 3). Additionally, we employed the EQ VAS, the vertical visual analog scale evaluated on a 0 to 100-point scale, which was derived using the 2018 Chinese version (49) to measure patients’ quality of life, where higher scores indicate a better quality of life.

#### 2.3.3 The problem areas in diabetes scale

Same as the original version (50), The Chinese version (51) PAID consists of 20 items rated on a Likert 5-point scale, ranging from 0 to 4, where 0 indicates no problem and 4 indicates a severe issue. To derive the total score (ranging from 0 to 100), all item scores are summed and multiplied by 1.25. A total score of 40 or higher suggests emotional distress related to diabetes management, warranting particular attention from healthcare providers.

#### 2.3.4 The appraisal of diabetes scale

ADS was created by Carey et al. (52), and was translated into Chinese by Li et al. (53). It is a seven-item self-evaluation scale that assesses the stress experienced by diabetic patients as a result of their disease. The scale employs a 5-point Likert scoring system, with 5 positively worded items and 2 negatively worded items. The total score is 35, and a lower score indicates that the patient views the disease more positively.

#### 2.3.5 The diabetes self-efficacy scale

The SED, created by Lorig et al. (54) and revised by Wei (55), comprises nine items across four categories in its Chinese version. Scored on a 5-point Likert scale, with 1 indicating a complete lack of confidence and 5 indicating complete confidence, the scale has a total score of 45. Higher scores signify greater confidence in blood glucose management.

### 2.4 Statistical analysis

All the below mentioned analyses were performed using Jamovi 2.4.8 and Mplus (version 7.4).

#### 2.4.1 Item analysis

Firstly, the top 27% of participants were placed in the high group and the bottom 27% in the low group after computing each participant’s overall score for all items. A *t*-test was used to compare the differences for each item between the high and low groupings. Secondly, to measure the correlation between each score and the overall score, Pearson’s correlation coefficient was computed. Thirdly, promax rotation was used to further screen items in an exploratory factor analysis (EFA). Prior to conducting the EFA, factor-ality was also evaluated using Bartlett’s test of sphericity and Kaise-Meyer-Olkin (KMO) measures.

#### 2.4.2 Reliability

We calculated Cronbach’s alpha coefficient and retest reliability.

#### 2.4.3 Validity and sensitivity

Confirmatory Factor Analysis (CFA) was performed on each of the three dimensions of the DHP18, and combined reliability and convergent validity were calculated from the factor loadings. We then conducted a test of variability of the DHP18 dimensions across subgroups of other variables to obtain the measurement sensitivity of the scale.

#### 2.4.4 Impacts of mHealth

Given that all the data in this study came from questionnaires, we used Harman’s single-factor test to control common method variance. Then, we calculated the correlation coefficients for every two variables. Additionally, mediation and moderation analyses will be employed to compute and model the correlation between the scores on the various diabetes-related models.

## 3 Results

### 3.1 Reliability

The results of the item analysis showed that all the DHP18 items differed at the level of significance on the high and low subgroups, and the correlation coefficients between each item and the total score were also greater than 0.40 and all reached the level of significance (*p* < 0.001). With a KMO value of 0.87 and a statistically significant Bartlett’s ball test ( $\chi^{2}$ = 3,215, *df* = 153, *p* < 0.001), the data were suitable for factor analysis. Table 2 illustrates the total Cronbach’s alpha coefficient and test–retest reliability, along with the corresponding values for each dimension of the DHP18. Both the total Cronbach’s alpha coefficient and coefficients of test–retest reliability of the questionnaire exceed 0.80, while each dimension demonstrates Cronbach’s alpha coefficient and coefficients of test–retest reliability surpassing 0.70.

**Table 2:** Cronbach’s alpha and coefficients of retest reliability.

Scale	Total scale	PD	BTA	DE
Cronbach’s *α*	0.88	0.84	0.76	0.81
Retest reliability	0.84	0.71	0.74	0.74

### 3.2 Structural validity

CFA was performed on the three dimensions of the DHP18 using Mplus (Version 8.1), and composite reliability and convergent validity were calculated using factor loading. Table 3 shows the results, with CR ranging from 0.77 to 0.85 for each dimension, all of which exceed the 0.60 minimum standard, indicating high reliability of the sample data in each dimension. However, in this study, the Average Variance Extracted (AVE) for the BTA dimension measured 0.33, falling below the factor loading standard of 0.36. Additionally, both the Tucker Lewis Index (TLI) and Comparative Fit Index (CFI) fit indices for the BTA were below 0.90 in the CFA model. These findings suggest that the convergent validity of BTA category of the scale in the present study was not satisfactory and that there is a need to consider further research to understand the necessity of this category and its related indicators.

**Table 3:** Validated factor analysis model fit indices for the sub-dimensional DHP18.

		Item	Factor loading	CR	AVE	CFA	
		TLI				CFI
PD	1. Lose temper over testing/diet	0.42	0.85	0.49	0.91	0.94
	2. Lose temper over small things	0.63				
	3. Touchy/moody about diabetes	0.78				
	4. Depressed because of diabetes	0.70				
	5. Lose your temper/shout due to diabetes	0.83				
	7. More arguments at home because of diabetes	0.76				
BTA	6. Avoid going out if sugars on the low side	0.50	0.77	0.33	0.76	0.84
	8. Food controls life	0.51				
	9. Edgy when out and nowhere to eat	0.74				
	10. Worry about colds and flu	0.60				
	11. Frightened in busy/crowded shops	0.54				
	17. Days tied to meal times	0.57				
	18. Difficulty staying out late	0.54				
DE	12. Wished not so many nice things to eat	0.42	0.82	0.48	1.00	1.00
	13. Eat something extra when bored	0.73				
	14. Not easy to stop eating	0.74				
	15. Eat to cheer self up	0.73				
	16. Hard saying no to food	0.78				

Given the Likert 4-point scoring system utilized by the DHP18 Scale, the MLM estimation method within Mplus was employed to address the possible bias derived from data’s non-normality. Residual correlations between specific items were permitted based on modification indices obtained through the MLM estimation method. The results of the first-order model for the recommended scale, outlined in Tables 4, 5, indicate that all models meet the specified standards.

**Table 4:** DHP validated factor analysis modified model fit indices.

Model	$\chi^{2}$	df	$\chi^{2}$ /*df*	CFI	TLI	RMSEA	SRMR	AIC	BIC
DHP-18	294.69	129	2.28	0.92	0.91	0.05	0.06	16348.07	16597.24

**Table 5:** DHP18 validated factor analysis modified model factor loading.

		Item	Factor loading		
		PD	BTA	DE
PD	1. Lose temper over testing/diet	0.45		
	2. Lose temper over small things	0.58		
	3. Touchy/moody about diabetes	0.78		
	4. Depressed because of diabetes	0.74		
	5. Lose your temper/shout due to diabetes	0.78		
	7. More arguments at home because of diabetes	0.72		
BTA	6. Avoid going out if sugars on the low side		0.47	
	8. Food controls life		0.51	
	9. Edgy when out and nowhere to eat		0.70	
	10. Worry about colds and flu		0.60	
	11. Frightened in busy/crowded shops		0.54	
	17. Days tied to meal times		0.58	
	18. Difficulty staying out late		0.55	
DE	12. Wished not so many nice things to eat			0.44
	13. Eat something extra when bored			0.74
	14. Not easy to stop eating			0.74
	15. Eat to cheer self up			0.71
	16. Hard saying no to food			0.77
	Factorial Correlation Matrix			
	BTA	0.68		
	DE	0.47	0.56	

### 3.3 Sensitivity

Table 6 presented the results of sensitivity and known-group validity. The DHP18 demonstrated limited differentiation between genders, educational levels, types of labor, housing situations, and marital status (indicating lower sensitivity) among social determinants. However, it notably showed effectiveness in distinguishing between patients in two different age groups, particularly in measuring PD. Moreover, DE was effective in differentiating between age groups, obese patients, and those within normal BMI ranges. All three subscales—PD, BTA, and DE—proved effective in distinguishing patients based on their ability to afford healthcare, indicating higher sensitivity in this aspect.

**Table 6:** Comparison of DHP18 subscales across social determinants.

Social determinants		*N*	Diabetes Health Profile-18 (DHP18)					
			PD		BTA		DE	
			*M* ± SD	ES*	*M* ± SD	ES*	*M* ± SD	ES*
Gender	Female	204	21.00 ± 17.90		17.10 ± 16.90		22.60 ± 21.30	
	Male	266	20.60 ± 17.80	−0.02	15.10 ± 15.30	−0.12	21.80 ± 20.40	−0.04
Age (years)	≤45	141	23.30 ± 17.90		16.50 ± 13.70		27.40 ± 20.50	
	>45	329	19.70 ± 17.70	0.20*	15.70 ± 16.90	0.05	19.80 ± 20.40	0.37***
Marriage	Single	18	18.80 ± 16.6		18.30 ± 11.20		28.90 ± 21.50	
	Married	411	20.80 ± 17.90		15.50 ± 16.20		21.80 ± 21.00	
	Divorced/widowed	36	22.50 ± 17.60	0.00	21.00 ± 16.10	0.01	21.50 ± 17.30	0.00
BMI (kg/m^2^)	<18.5	7	11.90 ± 11.80		27.90 ± 21.20		15.20 ± 13.70	
	18.5 ~ 24	135	20.00 ± 16.60		16.00 ± 16.10		19.30 ± 18.60	
	24 ~ 28	178	20.20 ± 17.70		15.10 ± 16.60		22.20 ± 20.80	
	≥28	147	22.80 ± 19.10	0.01	16.50 ± 14.80	0.01	24.90 ± 22.60	0.01
Capacity to pay for health services	No trouble	298	18.10 ± 16.00		14.00 ± 15.30		19.60 ± 19.50	
	Troubled	167	25.70 ± 19.90	−0.43***	19.80 ± 16.70	−0.36***	26.50 ± 22.30	−0.34***
Educational level	Junior and Lower	128	20.60 ± 18.10		17.20 ± 17.10		20.20 ± 20.90	
	Senior	168	20.60 ± 18.50		15.60 ± 17.00		20.80 ± 20.70	
	Three-year college	79	20.90 ± 16.90		16.70 ± 16.00		26.30 ± 20.70	
	Bachelors and Above	85	22.30 ± 17.40	0.00	15.50 ± 12.30	0.00	24.20 ± 20.70	0.01
Household	Live alone	46	20.90 ± 19.80		19.50 ± 17.40		24.80 ± 21.80	
	Not living alone	417	20.70 ± 17.60	0.01	15.60 ± 15.70	0.24	21.90 ± 20.70	0.14
Types of labor	Resting	49	23.60 ± 17.40		15.70 ± 15.70		19.30 ± 17.70	
	Light labor	352	19.90 ± 17.30		15.40 ± 15.30		21.80 ± 20.80	
	Medium to heavy labor	67	23.80 ± 20.40	0.01	19.30 ± 19.20	0.01	25.60 ± 22.10	0.01

^*^p < 0.05, ^**^p < 0.01, ^***^p < 0.001. ES, Effect Size; *M*, Mean; SD, Standard Deviation. Light labor refers to the physical intensity of types of work such as office workers, teachers, etc. Medium to Heavy Labor refers to the physical intensity of such types of work as drivers, waiters, construction workers etc.

Table 7 displayed the differential analysis results of DHP18 across various clinical and psychological determinants. Within clinical determinants, the DHP18 subscales exhibited limited capacity to differentiate between patients with varying diabetes durations and different drug treatment methods. Notably, in this survey, only the DE subscale effectively distinguished patients based on their regularity of medical reviews and blood glucose control, using an HbA1c level of 7% as the cutoff point. Regarding psychological determinants, the PAID scores, categorized into two groups based on a total score of 40 points, revealed significant differences in DHP18 subscale scores, indicating a substantial effect size between high and low PAID scores.

**Table 7:** Comparison of DHP18 subscales across clinical and psychological determinants.

Clinical determinants		*N*	DHP					
			Psychological distress		Barriers to activity		Disinhibite eating	
			*M* ± SD	ES*	*M* ± SD	ES*	*M* ± SD	ES*
Duration of diabetes (years)	<1	95	20.50 ± 18.40		14.80 ± 14.90		22.70 ± 22.10	
	1 ~ 5	214	21.30 ± 18.80		16.70 ± 16.40		22.90 ± 20.90	
	≥5	161	20.20 ± 16.00	0.00	15.70 ± 16.10	0.00	20.70 ± 19.70	0.00
HbA1C (%)	≤7	124	19.40 ± 16.80		14.70 ± 15.40		17.60 ± 23.60	
	>7	344	21.20 ± 18.20	−0.12	16.40 ± 16.20	−0.19	23.60 ± 21.20	−0.38***
Regular medical visits	Yes	121	20.90 ± 18.00		16.70 ± 17.00		19.60 ± 19.00	
	No	347	21.00 ± 17.90	−0.01	16.00 ± 15.50	0.04	23.70 ± 21.60	−0.20*
Diabetes related therapy	Oral medication	305	20.60 ± 17.30		15.60 ± 16.00		21.90 ± 21.30	
	Insulin	24	16.90 ± 16.30		19.00 ± 16.60		21.70 ± 19.50	
	Oral medication and insulin	139	21.90 ± 19.10	0.00	16.30 ± 16.00	0.00	22.60 ± 19.80	0.00
Psychological determinants								
PIAD score	≥40	47	39.40 ± 19.20		33.40 ± 18.00		40.60 ± 22.70	
	<40	398	18.70 ± 15.80	−1.27***	14.30 ± 14.60	−1.28***	20.20 ± 19.30	−1.03***

^*^p < 0.05, ^**^p < 0.01, ^***^p < 0.001. ES, Effect Size; *M*, Mean; SD, Standard Deviation.

### 3.4 Impacts of mHealth

The differences in blood sugar levels (measured by HbA1C) among patients receiving different types of health education were calculated based on the different health education groups. Table 8 showed that all forms of health education, particularly remote care, have a significant positive impact on blood sugar control.

**Table 8:** Effect of different forms of health education on blood glucose levels.

Diabetes education		*M*	SD	*t*	*p*
Not educated	mHealth education	2.26	0.22	10.16	***
	Other education	0.48	0.20	2.45	*
mHealth education	Other education	−1.78	0.24	−7.31	***

^*^p < 0.05, ^**^p < 0.01, ^***^p < 0.001. *t*, *t*-test score; *M*, Mean; SD, the Standard Deviation. Other health education includes all forms of health education on diabetes that did not use the remote tracking of the system in this study, e.g., nurse presentations in outpatient clinics or wards, small group teaching, look-and-talk, etc.

Table 9 showed that all DHP subscales distinguished well between general diabetes health education (professional talk on diabetes self-management) and mHealth education. The mhealth education included a multidisciplinary team of physicians, nurses, health educators, and dietitians provided continuous, real-time, individualized healthcare through a mobile-based intervention on glycemic control in patients with T2DM. However, no substantial variances in BTA and DE categories were observed between patients who had never received health education and those who were educated online. Furthermore, no statistically significant differences were found between patients who had never received diabetes self-management health education and those who had, with minimal effect sizes observed (PD: *p* = 0.69, ES = 0.04; BTA: *p* = 0.07, ES = 0.19; DE: *p* = 0.26, ES = 0.12).

**Table 9:** Comparison of DHP18 subscales across different forms of health education.

Clinical determinants		*N*	DHP18					
			PD		BTA		DE	
			*M* ± SD	ES*	*M* ± SD	ES*	*M* ± SD	ES*
Diabetes education	Not educated	234	21.50 ± 17.90	−0.27*	15.70 ± 16.40	−0.21	22.40 ± 20.80	−0.24*
	mHealth education	95	16.80 ± 16.10		12.50 ± 12.90		17.60 ± 16.10	
	Other education	141	22.30 ± 18.50	−0.31*	18.80 ± 16.80	−0.42**	24.80 ± 23.00	−0.35**

^*^p < 0.05, ^**^p < 0.01, ^***^p < 0.001. ES, Effect Size; *M*, Mean; SD, Standard Deviation. Other health education includes all forms of health education on diabetes that did not use the remote tracking of the system in this study, e.g., nurse presentations in outpatient clinics or wards, small group teaching, look-and-talk, etc.

We examined an unrotated EFA solution with a single-factor solution. The first factor loading was 31.89%, which is less than the generally adopted critical standard of 40%. This result indicated the suitability of following mediation and moderation analysis. Table 10 showed the correlations between the different scale dimensions in this study.

**Table 10:** Correlations between ADS, SED, EQ-5D-3L and the three dimensions of the DHP18.

	PD	BA	DE	EQ-5D	EQ-5D VAS	PAID	SED	ADS
DHP18								
PD	–							
BA	0.57***	–						
DE	0.45***	0.54***	–					
EQ-5D-3L								
EQ-5D	0.32***	−0.33***	−0.21***	–				
EQ-5D VAS	−0.32***	−0.29***	−0.24***	0.38***	–			
PAID	0.57***	0.62***	0.51***	−0.36***	−0.37***	–		
SED	−0.31***	−0.24***	−0.31***	0.21***	0.41***	−0.35***	–	
ADS	0.45***	0.44***	0.36***	−0.33***	−0.31***	0.67***	−0.35***	–

*p < 0.05, **p < 0.01, ***p < 0.001. EQ-5D is the utility score value obtained using the quality of life utility score calculation method for the 2018 China EQ-5D-3L; EQ-5D VAS is a visual direct assessment by patients of their current state of health-related quality of life; PAID is the total score of the Problem Areas in Diabetes Scale; SED is the total score of the Diabetes Self-Efficacy Scale; ADS is the total score of the Appraisal of Diabetes Scale.

**Figure 1:**
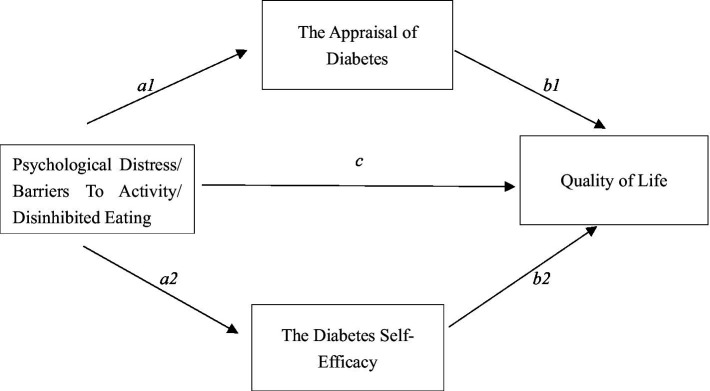
Mediation modeling of DHP18 dimensions on subscales. a1 and b1 are specific indirect effect 1, a2 and b2 are specific indirect effect 2, c is direct effect.

As shown Figure 1, the mediating effects of ADS and SED on the impact of Subscales in the DHP18, including PD, BTA, and DE, on the direct assessment of HRQOL in the EQ-5D-3L VAS were analyzed.

The results are shown in Table 11. The three DHP18 variables significantly underpredicted patient EQ-5D-3L VAS scores for ADS, SED, and QoL. According to the findings of the mediation analysis, ADS and SED were the key mediators in the significant direct and indirect effects of PD and BA on Qol. The BA path was responsible for 49.15% of the total effect, with the PD path’s mediating effect accounting for 47.47% of it.

**Table 11:** Mediating effects of ADS, SED on the three dimensions of the DHP18.

	Point estimate	Product of coefficients			BC bootstrap 1,000 times 95% CI	
		S.E.	Est./S.E.	*p*-value	Lower	Upper
Psychological Distress (PD)						
Total effect	−0.26	0.04	−5.92	***	−0.35	−0.18
Total indirect effect	−0.12	0.03	−4.93	***	−0.18	−0.08
Specific indirect effects 1	−0.05	0.03	−2.06	*	−0.11	−0.01
Specific indirect effects 2	−0.07	0.02	−3.57	***	−0.12	−0.04
Direct effect	−0.14	0.04	−3.19	**	−0.22	−0.05
Difference in indirect effects	0.02	0.04	0.53	0.60	−0.05	0.10
Barriers to Activity (BTA)						
Total effect	−0.23	0.05	−4.91	***	−0.34	−0.16
Total indirect effect	−0.12	0.03	−4.28	**	−0.17	−0.07
Specific indirect effects 1	−0.06	0.03	−2.33	*	−0.12	−0.01
Specific indirect effects 2	−0.06	0.02	−2.79	**	−0.11	−0.03
Direct effect	−0.12	0.05	−2.47	*	−0.22	−0.03
Difference in indirect effects	−0.00	0.04	−0.12	0.90	−0.08	0.08
Disinhibited eating (DE)						
Total effect	−0.13	0.02	−3.25	***	−0.20	−0.07
Total indirect effect	−0.10	0.02	−4.87	***	−0.15	−0.07
Specific indirect effects 1	−0.05	0.02	−2.74	**	−0.07	−0.02
Specific indirect effects 2	−0.06	0.02	−3.25	**	−0.10	−0.03
Direct effect	−0.03	0.03	−0.89	0.38	−0.09	0.04
Difference in indirect effects	0.01	0.03	0.25	0.80	−0.05	0.07

^*^p < 0.05, ^**^p < 0.01, ^***^p < 0.001. Specific indirect effect 1 was the mediating effect of ADS on the Qol of patients for each variable in the DHP18, and specific indirect effect 2 was the mediating effect of SED on the Qol of patients for each variable in the DHP.

Results of the mediation effect testing model show that the overall effect (*β* = −0.13, *p* < 0.001) and the indirect effect (*β* = −0.10, *p* < 0.01) of DE on Qol are both significant, but the direct effect is not significant (*β* = −0.03, *p* = 0.38). The complete mediating effect of ADS and SED between these two variables is significant, with a total mediation rate of 77.61%, of which ADS accounts for 36.57% and SED accounts for 41.04%. The impact of dietary restraint on Qol is achieved entirely through the indirect pathways of ADS and SED.

## 4 Discussion

This study validated the effectiveness of the Chinese version of DHP18 and identified some key factors affecting the quality of life of T2DM patients, such as age and economic conditions. Notably, mHealth interventions exhibited a positive impact on the quality of life while also enhancing glycemic control, shedding light on the potential reasons for this favorable outcome.

In this study, the validation of the DHP18 demonstrated its satisfactory validity, reliability, and sensitivity in gaging the psychological well-being of individuals with T2DM. It also had a high criterion validity with another well-studied diabetes-specific distress scale (PAID). We discovered, however, that the BTA subscale has a poor convergent validity, which was consistent with the scale’s validation results in Singapore and Ecuador (44, 45) but differs from its original finding in UK, indicating the possible lacking of the cross-cultural consistency of the structure of DHP18.

Regarding the Effect Sizes (ES), younger diabetic patients (under 45 years) experienced higher psychological distress and greater dietary restriction. Challenges such as availability of healthy meals at work, juggling regular meals and medication, and managing self-health assessments in demanding job roles and the more frequent complications (56) exacerbated stress for younger individuals. Studies indicate that eating behavior might alter under stress (57), potentially explaining the larger effect sizes observed in the DE category (45), which is also applicable to young diabetic patients in China. Moreover, the affordability of diabetes treatment demonstrated significant effect sizes across all three DHP18 dimensions. Limited financial resources correlated with heightened psychological distress (58), impacting behavioral control and dietary management.

Moreover, another important aim of this study is to reveal the underlying mechanism of the mHealth model’s positive function in promoting diabetic patients’ QoL. We highlighted patients’ self-evaluations of diabetes stress and self-efficacy as mediators, and found self-evaluations (59) and self-efficacy (60) can fully mediate the effects of DE on improving patients’ QoL. These results indicate psychological distress, behavioral restrictions and dietary stipulations’ nonnegligible impact on diabetes management. Further analysis on mHealth data showcased the benefits of any form of health education in improving glycemic indicators. Traditional health education often lacked effective measures to monitor patients’ behavioral changes and misconceptions, while the mHealth diabetes management model (34), integrating offline education and remote tracking, empowered patients to apply diabetes knowledge effectively, resolving the misconceptions. The results showed that patients’ own dietary habits have no impact on quality of life; while it is the dietary changes required to manage blood glucose, the inconvenience of living with the behavioral demands of blood glucose self-management, and the emotional distress of diabetes that affect the patients’ quality of life. The usage of mHealth for blood glucose management can provide immediate technical support to alleviate negative emotions from these three areas of DHP18, resulting in positive improvements in self-efficacy and diabetes self-assessment for T2DM patients thus improving their quality of life.

### 4.1 Implications

This study provides a new Chinese version of the Multidimensional Diabetes-Related Quality of Life Scale for diabetes in China to help healthcare professionals to better tailor mental health education for T2DM patients. Moreover, this study reminds diabetes health educators to focus on special populations and enhance psychological screening for special groups, such as young adults and those with high economic stress. In addition, our findings suggest that online medical education and healthcare play a positive role. The role of mHealth is to curb incorrect glucose control habits more efficiently and accurately. Real-time remote professional guidance from diabetes healthcare professionals can encourage patients to develop a positive concept of diabetes self-management, increase self-efficacy, and improve their evaluation of diabetes, thus improving their quality of life.

### 4.2 Limitations and future research direction

This study has several limitations. Although the DHP-18 can be used in patients with type 1 or T2DM, the psychometric test was not performed in patients with type 1 diabetes, so the results are only applicable to patients with T2DM. Secondly, due to the cross-sectional nature of this study, there was no cohort follow-up to understand patients’ ongoing quality of life. Thirdly, we investigated patients in only one hospital in Tianjin, China, thus limiting the generalizability of our findings. Multi-center survey and cohort follow-up study are suggested to further prove the psychometric properties and its correlation with other important variables predicting the health condition of patients with T2DM.

## 5 Conclusion

1. The Chinese version of Diabetes Health Profile Scale has good measurement properties and is appropriate for measuring HRQL in Chinese patients with T2DM.
2. Diabetes health education and support in the mobile health model plays a positive role in the quality of life of people with T2DM.
3. The mHealth model can play a role in improving the quality of life of patients with T2DM by increasing their sense of efficacy in blood glucose self-management and improving their self-assessment of having diabetes.

## Data availability statement

The raw data supporting the conclusions of this article will be made available by the authors, without undue reservation.

## Author contributions

XL: Funding acquisition, Writing – review & editing. JZ: Data curation, Formal analysis, Investigation, Writing – original draft. JL: Resources, Supervision, Writing – review & editing. YS: Data curation, Writing – original draft. TY: Data curation, Writing – original draft. WH: Data curation, Writing – original draft.
